# The social value of clinical research

**DOI:** 10.1186/1472-6939-15-66

**Published:** 2014-09-05

**Authors:** Michelle GJL Habets, Johannes JM van Delden, Annelien L Bredenoord

**Affiliations:** 1 Department of Medical Humanities, Julius Center for Primary Care and Health Sciences, University Medical Center Utrecht, Heidelberglaan 100, 3584 CX Utrecht, The Netherlands

**Keywords:** Social value, Ethics of translational medicine, Research ethics

## Abstract

**Background:**

International documents on ethical conduct in clinical research have in common the principle that potential harms to research participants must be proportional to anticipated benefits. The anticipated benefits that can justify human research consist of direct benefits to the research participant, and societal benefits, also called social value. In first-in-human research, no direct benefits are expected and the benefit component of the risks-benefit assessment thus merely exists in social value. The concept social value is ambiguous by nature and is used in numerous ways in the research ethics literature. Because social value justifies involving human participants, especially in early human trials, this is problematic.

**Discussion:**

Our analysis and interpretation of the concept social value has led to three proposals. First, as no direct benefits are expected for the research participants in first-in-human trials, we believe it is better to discuss a risk- value assessment instead of a risk - benefit assessment. This will also make explicit the necessity to have a clear and common use for the concept social value. Second, to avoid confusion we propose to limit the concept social value to the intervention tested. It is the expected improvement the intervention can bring to the wellbeing of (future) patients or society that is referred to when we speak about social value. For the sole purpose of gaining knowledge, we should not expose humans to potential harm; the ultimate justification of involving humans in research lies in the *anticipated social value* of the intervention. Third, at the moment only the validity of the clinical research proposal is a prerequisite for research to take place. We recommend making the anticipated social value a prerequisite as well.

**Summary:**

In this paper we analyze the use of the concept social value in research ethics. Despite its unavoidable ambiguity, we aim to find a best use of the concept, subject to its role in justifying involving humans in first-in-human research.

## Background

International documents on ethical conduct in clinical research have in common the principle that potential harms to research participants must be proportional to anticipated benefits. In first-in-human trials, participants are not expected to gain a direct benefit as the purpose of those trials is to examine the safety of the intervention, and often non- therapeutic dosages are used. Although a direct benefit cannot be excluded in phase I trials, they are not the purpose of the study and should therefore not play a role in the evaluation. This is especially evident when healthy volunteers are used for these early human trials

In the absence of direct benefits, risks have to be proportional to the value the study has for society. The concept social value is assumed to be sufficiently clear that it can function in discussions on the justification of research. Indeed, we all have an implicit notion of what is meant by social value; most people will agree that cancer research has social value; less agreement may exist on whether research into a human expedition to Mars will have social value. However, social value is used in many different ways in our everyday use, in the social sciences, and also in the academic literature on research ethics. Although the concept is ambiguous, few critical reflections on the concept in research ethics have been completed, despite the justifying role it plays in (early human) clinical trials [1-5]. As risks are highly unpredictable in first-in-human trials, it seems problematic to uncritically use an ambiguous concept to justify risks to research participants. Here, we will first aim to clarify some of the causes of the confusion in the concept ‘social value’. Despite its inherent problems, we will next propose to limit social value to refer to the anticipated improvement the intervention will bring, which is what we believe would be the best use of the concept. This is important for we deem the anticipated social value of the intervention should be a prerequisite for research to involve human participants. Last, we will briefly discuss who should be responsible for assessing the anticipated social value of medical interventions. We aim to provide a foundation for further discussion.

## Discussion

### Plurality of social value concepts in research ethics

The concept social value is used in different ways in the academic literature in research ethics and various terminologies have been used to refer to what we call social value, like e.g. the importance of research, the relevance of research, humanitarian value, clinical value and health value. Only few articles explicitly describe the social value of research [1,2,4-7]. Here we will give a brief overview of some of these articles to show that even when social value is the object of inquiry, there are still numerous ways in which it is used.

According to the eight principles of “What makes research ethical”, the social value of research is the ultimate improvement in health.

“Clinical research has instrumental value because it generates knowledge that leads to improvement in health. It is *such improvements* that ultimately constitute the social value of research” [8].

Knowledge value and social value are thus distinguishable according to Emanuel and colleagues, and it is the improvement in health that gives research instrumental value.

In contrast, guideline 8 of CIOMS’ International Ethical Guidelines for Biomedical Research Involving Human Subjects does not appear to discriminate between benefits to society and generalizable knowledge [9].

Risks or interventions that do not hold out the prospect of direct diagnostic, therapeutic or preventive benefit for the individual must be justified in relation to the expected benefits to society (generalizable knowledge) [9].

For the CIOMS guidelines, the knowledge research provides, can be *extrinsically* valuable (it has value for the sake of something else that is good), but also intrinsically valuable (value a thing has in itself). Cassarett and colleagues also assign value to *knowledge*. According to them: “A central goal of research is to produce knowledge that is “important”, “fruitful” or that will have value” [4].

According to Kimmelman, social value can be assigned to *clinical trials*, but also to the *information* human experiments produce [5]. In addition to assigning value to different objects, Kimmelman also distinguishes between different kinds of value, like progressive value (the value of a trial is seen as the likelihood it will progress to the next phase), translational value (the value of a trial is perceived to be wider than merely progression to the next phase, and also includes informing preclinical testing, other areas of research, or a change in the intervention), and humanitarian value of clinical trials (the value clinical research has because ‘it advances broader societal ends like improved healthcare’) [5]. Casarett and colleagues distinguish two categories in their proposed term *health value* (the potential of a study to improve health): *immediate* health value and the *future* health value of a study [4]. Examples of studies that have immediate health value are phase III clinical trials as they have the potential to improve health as soon as the drug has been proven effective and approved. Phase I trials, in contrast, have potential future health value as their value lies in their contribution to future research. Karlawish makes a similar distinction when he claims that research that will directly change clinical practice has *clinical* value, which is optional for a study, whereas scientific validity and value is necessary and sufficient [2]. (In this paper we will use first-in-human and phase I studies interchangeably, although we are aware that first-in- human research consist of more than merely phase I trials.)

We question whether they are really distinguishing between kinds of value clinical trials can have, or whether they are pointing to different things we value in trials. We also question whether distinguishing between different objects that can have social value, *and* distinguishing kinds of values, really makes transparent which clinical trials are worth doing.

In addition, in the specific case of Karlawish and Casarett, where two categories are important in balancing a study’s risks and values, (value and clinical value; immediate health value and the future health value resp.) an inconsistency can be found. According to them, a phase III is more likely to have clinical value than a phase I. Early human studies, such as phase I trials, can get stuck in the so-called Valley of Death: “the widening gap between advances in basic science and the practical application of that knowledge” [10]. In phase III studies, safety and efficacy has already been examined and they are thus more likely to provide knowledge that may lead to clinical improvement. But, if the value of phase I is lower (because it either has no *clinical value* or because it has no *immediate health value*), as it merely contributes to future research, why do we accept higher risks here? Indeed, a phase I trial is performed to *examine* the risks; moreover, often, healthy volunteers are enrolled, who thus will not benefit.

We believe that the assignment of ‘knowledge value’, ‘clinical value’, ‘progressive value’, or ‘future health value’ to *research*, *knowledge, information*, *clinical trials* and the *intervention* leads to confusion as to what exactly *has* social value (leaving aside the question what *is* social value). We believe it is important to be clear on the exact meaning of social value precisely because it is used to justify involving human research participants in first-in-human trials. Simultaneously, we are aware of the fact that the ambiguity of the concept value will necessarily cause confusion, as we will show below.

### Unravelling some of the difficulties of ‘social value’

The concept ‘value’ has been under scrutiny in many different research fields like sociology and philosophy, however, little agreement exists on even the definition of value. Consent *does* exist on the fact that values arise out of human experience [11]. Whereas the term ‘benefit’ refers to an advantage or profit gained from something, the concept value refers to the ‘regard that something is *held* to deserve’ [12]. The latter is thus a relational concept; both the object to be valued, and an evaluator are necessary preconditions for value to exist. Flowers can e.g. benefit from rain, but rain is not *valuable* to flowers. It is precisely this act of appraisal that makes an analysis of (social) value difficult.

In our everyday use, the concept of social value can function in two main ways. First, social values can be seen as values shared by a community of individuals; they are values held by society and are contrasted with individual (non-shared) values. “By social values we refer to socially collective beliefs and systems of beliefs that operate as guiding principles in life […]” [11]. Second, besides values *of* society, the concept can also be used to refer to values *for* society. Here, social value is an assigned predicate or property of an object, [13] and in our case, of research. This distinction reflects the conundrum of what is primary: values or valuing? Is a thing valuable because it is valued, or is it valued because it is valuable? [14] This difference can also be perceived when we consider the questions: ‘is this intervention valuable for society?’ and “will society value this intervention?” In the latter case, we do not have to make calculations, but merely need to look at the emotional attitude ‘society’ has; in the former, we need to estimate whether it will be valuable for society, whether it will benefit society [15]. A first step in clarifying the concept social value would be to distinguish these uses of the concept; however, whether we believe something is valuable to society is dependent on the moral values we collectively share, or the standards we have for evaluating. There is thus a certain circularity involved, which adds to the confusion. Unfortunately, it does not lie in the scope of our paper to extensively analyse the concept ‘value’, nor do we think we could solve what so far has not been solved in many different fields. Because of the inherent confusion of the concept value we might choose to abandon ‘social value’ from research ethics altogether; however, it is questionable whether a replacement for it will be any less confusing. Indeed, the extra dimension that social value adds to a mere benefit or advantage, namely the importance, appreciation, or worth, captures exactly that which makes it a confusing concept. Trying to discard the problems inherent to using the term social value would entail disposing human evaluation, which would be throwing out the baby with the bathwater.

### The anticipated social value of medical interventions

In the remainder of this paper, we will make suggestions on how the concept social value can function in clinical research settings. First, we propose to speak of a risk-value assessment in phase I trials instead of a risk- benefit assessment, because no direct benefit to the research participant in expected, and social value needs to be evaluated in order to justify the trial. Although we are aware that participants *can* benefit from first-in-human trials, [16] this is irrelevant, since the evaluation and justification of a trial needs to precede the initiation of the trial, and phase I trials are designed for safety and feasibility, not to provide direct benefits. Whether phase I trials *should* offer a direct benefit is an important but different question that unfortunately does not lie in the scope of our paper [17].

Second, we therefore propose that the social value (*for* society) of an intervention lies in the nature and magnitude [6] of the improvement the intervention is expected to have on the wellbeing of patients, individuals in society, or society (but not research participant, for this we would call a direct benefit!). This we will call the *anticipated social value* of the intervention. (We will use the concept “intervention” to refer to both *what* intervenes (the drug, cell line, tissue, device, etc.) ánd *how* it intervenes, or the *application* of the drug, cell line, tissue *etc*. “Intervention” is derived from the Latin word *intervenire,* which means “to come between”, “to step in”; the application is thus integral in our concept “intervention”). We will not ascribe anticipated social value to clinical trials testing the intervention, nor to knowledge generated by clinical trials (although the application in the clinic resulting from the knowledge gained, can have social value). Although we do not deny that humans value knowledge as well as research, for justification of exposing human participants to risk, this *valuing of* is not enough. Ultimately, it is the intervention or the application of the knowledge that will have value *for society*. Research is a means to this end: research is done in order to gain knowledge. Moreover, in clinical trials when humans are exposed to risks, justification needs more than the mere gain of knowledge. When we speak about value *for* society, we prefer to only refer to the improvement the intervention is likely to have on the wellbeing of patients. Of course, our proposal will have limitations, because ‘value’ will always be ambiguous in its use, but limiting its use will be a clarification. A major advantage to our proposal is that a clinical trial itself cannot justify involving humans, despite its possible perfect design, and likely progression to the clinic.

Two others questions important for the evaluation of clinical trials are: i) will the trial likely be successful in facilitating the introduction of the intervention to society? In other words, will this specific research (design) likely progress the transition of the device or intervention to the clinic –whether from preclinical research or phase III research? This we will call the *translational prospect* of a clinical trial because it specifically addresses the *likelihood* a clinical trial will assist in the translation of the intervention (and thus not its value). The other question is: ii) will the trial (design) be able to answer the research question? This has been called the validity of research.

To reiterate; in early human clinical studies there are three main assessments; the anticipated social value of the *intervention*, the translational prospect of the *clinical trial*, and the validity of a *study design*. For phase II and III, direct benefits to the research participant also need assessing. To clarify the distinctions, I will give an example of a clinical trial with limited translational prospect of an intervention with high anticipated social value. In the nineties, a Phase III clinical trial took place, testing a new antimicrobial peptide for efficacy in treating mildly infected foot ulcers of diabetics [18]. Because of an increase in antibiotic resistance worldwide, finding an efficacious drug alternative for antibiotics is an important objective. Another advantage is that because the drug is topical, it may have fewer adverse side- effects than systemic antibiotics. We could thus have assigned anticipated social value to the new intervention. The translational prospect of this trial turned out to be low because of the chosen comparator. The primary outcome measure was the comparison of a reduction in clinical signs and symptoms of infections between the new drug and an antibiotic. However, the used antibiotic was not indicated for infected foot ulcers, nor was the efficacy of using any antibiotic for mildly infected foot ulcers ever proven. It may be, that a placebo control (standard wound care) would have been as efficacious as the new antimicrobial peptide. The comparator was thus not well chosen, and the FDA required an additional placebo-controlled trial. Although the primary outcome measure was reached, showing equivalence did not move forward the translation of the antimicrobial peptide (we are leaving aside here the ethical question of using a placebo). Other reasons why a trial may not move forward the translation of an intervention is e.g. imperfectly chosen clinical endpoints, or (foreseen) non-compliance in the control group. The validity of a trial needs to be judged by an expert methodologist, and is more straightforward than both the anticipated social value and the translational prospect.

It will be clear from this example, that if an intervention is likely to increase the wellbeing of (future) patients it will do so regardless of the phase of the research. This does not mean that the anticipated social value cannot change during the translation because of new knowledge, or because of a change in environmental factors. For example, the heart mate was initially meant to temporarily help patients waiting for a heart transplant, but is now also used *instead of* heart transplantation. Due to an increase in knowledge on the efficacy of the heart mate, the anticipated social value would have increased during the trial. A change in environmental factors can have a similar effect. For example, the anticipated social value of the intravenous TKM 100201 infusion for the treatment of Ebola would have been assessed differently in 2011, than it would be now, due to the outbreak of Ebola in Africa. It is also possible that the intervention itself changes: Sildenafil, a drug studied for angina pectoris and hypertension, was found to induce penile erections during the first-in-human trials. The social value thus changed from what it was anticipated to be, but this is merely a result of a change in the intervention. It is no longer an intervention against hypertension, but it became an intervention (also) for erectile dysfunction. Although the anticipated social value can change, in general, large changes are not expected.

In contrast, the translational prospect of a clinical trial does change between phase I, II and III. Undeniably, a phase III trial is more likely to lead to the translation of the intervention to society than a phase I; however, this is essentially trivial. The question: will the trial be successful in bringing the intervention closer to the bedside, has to be evaluated *within* the phase of the study. The validity of a trial needs to be assessed for each trial and is thus by necessity different; indeed, the validity of a phase I trial cannot be said (in general) to be lower than the validity of a phase III trial.

Our proposal to remove ‘likelihood of translation’ out of the value- concept, will not lead to the inconsistency of phase I trials having lower value and higher risks. However, this does not mean that we avoid the problem of justifying these higher risks in first-in-human studies. For there *are* higher risks, and in contrast to phase II and III, the direct benefits to participants are unlikely.

### The anticipated social value as prerequisite for research involving humans

At present, none of the major international documents [9,19-21], including the recently revised Declaration of Helsinki, mention social value. However, they do all refer to for example, the importance or relevance of research.

In the Nuremberg Code, the relevance of research is a precondition for research to be ethical [9,14]. Indeed, paragraph 2 of the Nuremberg code reads:

“The experiment should be such as to yield fruitful results for the good of society, unprocurable by any other methods or means of study, and not random or unnecessary in nature” [21].

In the Declaration of Helsinki, in contrast, the importance of research is merely a component in the risks/benefit or value analysis:

“Medical research involving human subjects may only be conducted if the importance of the objective *outweighs* the inherent risks and burdens to the research subjects” [20].

The Declaration of Helsinki does thus not necessarily prevent futile research from taking place. The relevance of research is merely a component in the risk-benefit calculation. Whether or not research is ethical therefore depends on a balancing exercise. For the Nuremberg Code this balancing will also take place, but in addition, it has relevance as a minimal requirement [1]. This difference between Nuremberg and the Declaration of Helsinki should not be mistaken for a trivial distinction. Nor should one underestimate the amount of clinical studies that fail to improve the wellbeing of (future) patients it is supposed to improve: indeed, more than 75% of the 1000 new drugs approved by the FDA from 1990–2002 had a similar therapeutic quality as existing drugs [7]. The studies into those drugs *could* have been unethical according to the Nuremberg Code, however, not according to the Declaration of Helsinki.

Although validity is an accepted prerequisite for research to happen, the discussion on whether the anticipated social value is a necessary requirement still needs taking place. According to Freedman, the Helsinki model is preferred, as he sees no advantage in judging value prior to a risk-benefit analysis, especially because the value *of a study* needs to be judged “within the context of *all* other elements of the ethics of research” [1]. Only validity should be (and is at the moment!) a prerequisite. In Switzerland, a new law has come into force in 2014, requiring research -involving humans- to aim to answer “a relevant research question” [22]. Here, social value thus becomes a prerequisite of human research, [23] which is coherent with the Nuremberg code.

We believe that for any inclusion of human participants in clinical trials, the anticipated social value of the intervention should be a precondition. An objection could be that certain medical discoveries have been made by coincidence, and that limiting clinical trials thus may limit medical advancement. We disagree as accidental findings cannot be planned, nor does it mean that we will not find any accidental findings anymore by restricting clinical trials to interventions with anticipated social value. Moreover, if research is unethical, it should not be performed, regardless of whether it used to be done, or whether it may lead to accidental scientific advances. To be clear, we are not arguing against basic research; our ‘framework’ only applies to research using human participants.

Two comments need to be made before we continue to the next section. First, as will be clear by now, despite the title suggests, we do not attribute social value to clinical research, but social value is the improvement the intervention is anticipated to bring to the wellbeing of patients and society. And second, although we have talked about social value within the paradigm of phasing research, this does not mean we adhere strictly to the phasing. However, also in different research paradigms, anticipated social value will be attributed to the intervention tested.

### Research funding agencies, ethical committees and the researcher

Yarborough suggests stating the (anticipated) social value in informed consent forms [7]. This would allow research participants to make an autonomous decision on whether they think the anticipated value of the intervention is worth entering a trial. We agree that the anticipated social value should be made transparent, however, just like ‘a volunteer is not free to accept any risk whatsoever’ [24], we deem a volunteer should not be free to accept partaking in a trial testing an intervention lacking anticipated social value. It is important that participants are aware that these trials are not conducted for their benefit, but for the benefit of society, and this should be clearly stated in the informed consent form so they can make an informed decision on whether to partake in the study.

Research Ethical Committees (REC) have the responsibility to protect the research participant from harm. They are the gatekeepers that should disapprove research proposals on interventions without anticipated social value. Although REC members have this responsibility, this does not mean they alone need to identify the anticipated social value of an intervention; this should both be done at the level of funding (with participation of the public) and the level of RECs (with voice to the research participant and the patient).

Whether a particular research *direction* has sufficient ‘value’ is at the moment decided by the research funding agencies, steered by political decisions. Although the public in a democracy has thus an indirect voice in the research agenda, it can be questioned whether there is enough transparency in the decisions made. Funding agencies determine priority by constructing research programs, within which calls for grant proposals are made. At present, funding agencies do not explicitly examine the anticipated social value of particular research projects *in view of the justification of using human participants*. Indeed, at the level of funding, the question whether the anticipated social value of an intervention is ‘large enough’ (brings a significant improvement in the wellbeing of (future) patients)) for humans to be justifiably exposed to possible risks is likely not explicitly addressed. In general, it is scientists, by peer review that judge research proposals, often on innovative elements, scientific validity, and track record of the scientists. We believe that it is important that society at large is involved in determining the anticipated social value of an intervention, especially as there is no balancing that can be done when anticipated social value is determined outside of a risk-benefit analysis. There is thus no standard to decide when an intervention has (enough) anticipated social value. What contributes to anticipated social value? Is severity of disease important, or number of patients suffering from the disease, expected improvement in quality of life, or fair innings? These are decisions that should be taken by the public at large.

The specific research proposal (in contrast to the research direction) needs to be judged by RECs by two decisions [1]. First, they need to address the question: does the intervention have anticipated social value? This they will not do unaided: researches should submit a document on the anticipated social value of an intervention. This document needs to be made by careful consideration of the information of researchers and patient representatives on the intervention. A meeting between researchers and patients moderated by someone independent, is one of the ways this can be done [25]. An advantage of this method is that researchers are forced to explain precisely what the anticipated social value of the intervention would be; to which problem it is a solution, and how it should be used. Patients can then through e.g. the card method [26] reflect on the effect of the intervention on their life, but also on society [25]. The card-based communication method has proven to aid patients join in a creative debate on the influence a new intervention will have on their life, and society, without having expert knowledge. Such a meeting could be a prerequisite for REC evaluation, although we are aware that there is already resistance against the enormous paperwork necessary for REC approval. However, if we want to take patient participation seriously, it will necessarily require additional work.

If RECs decide the intervention tested has anticipated social value, then they will have to make a risk-benefit assessment for phase II and III trials, or a risk-value assessment for first-in-human trials. If the research question will make a significant contribution to the progress of the intervention into the clinic, then more risks can be allowed than if the research question is less useful, or the validity of the trial not as waterproof. Here, for early human trials, the validity, translational prospect, and the anticipated social value are all weighed against the risks the research participant would take. But weighing only takes place in the second decision RECs should take.

## Summary

Although the concept social value is by nature ambiguous, and confusion may be hard to avoid, we have proposed to employ the concept ‘anticipated social value’ in research ethics to refer to the nature and magnitude of the improvement an intervention is expected to have on the wellbeing of patients. Research is the instrument or process of progressing an intervention from bench to bedside. We believe it is important to be meticulous in using ‘social value’, because in first-in-human studies, the anticipated social value justifies exposing the research participants to risks as no direct benefit for the participant is expected, which is especially evident when healthy volunteers are used for first-in-human trials. Therefore, we also propose to speak of a risk-value assessment for first-in-human trials instead of a risk-benefit assessment.

Distinguishing anticipated social value of the intervention from the translational prospect and validity of research may prevent the inconsistency which exists for most authors that have attempted to make a value taxonomy, which is that if research itself has value (social value, clinical value, or translational value), and phase III research has more value than phase I, it seems hard to defend why we accept more risks in phase I.

We believe that the anticipated social value should be a prerequisite for first-in- human research to take place, as it is already the case for the validity of research. RECs should therefore make two evaluations for first-in-human trials. First, they need to consider whether the intervention tested has anticipated social value. If not, they should reject the proposal; if the intervention does have anticipated social value, they need to weigh the anticipated social value together with the validity and translational prospect (and possible direct benefits) against the risks the research participants will be exposed to. Whether an intervention has anticipated social value is something that needs to be decided by specialists, like researchers and RECs, but also by patients, and the public, through politics and funders.

## Competing interests

The authors declare that they have no competing interests.

## Authors’ contributions

All authors participated in the design of the study. MGJLH drafted the manuscript. All authors made substantial contribution to the manuscript and revised it critically. All authors have read and approved the final manuscript.

## Pre-publication history

The pre-publication history for this paper can be accessed here:

http://www.biomedcentral.com/1472-6939/15/66/prepub
